# Hormonal Contraceptives and Cerebral Venous Thrombosis Risk: A Systematic Review and Meta-Analysis

**DOI:** 10.3389/fneur.2015.00007

**Published:** 2015-02-02

**Authors:** Farnaz Amoozegar, Paul E. Ronksley, Reg Sauve, Bijoy K. Menon

**Affiliations:** ^1^Department of Clinical Neurosciences, University of Calgary, Calgary, AB, Canada; ^2^Department of Clinical Epidemiology, Ottawa Hospital Research Institute, Ottawa, ON, Canada; ^3^Department of Pediatrics and Community Health Sciences, University of Calgary, Calgary, AB, Canada; ^4^Department of Radiology, University of Calgary, Calgary, AB, Canada; ^5^Hotchkiss Brain Institute, Calgary, AB, Canada

**Keywords:** hormonal contraceptives, birth control pill, oral contraceptive pill, cerebral venous sinus thrombosis, cerebral venous thrombosis

## Abstract

**Objectives:** Use of oral contraceptive pills (OCP) increases the risk of cerebral venous sinus thrombosis (CVST). Whether this risk varies by type, duration, and other forms of hormonal contraceptives is largely unknown. This systematic review and meta-analysis update the current state of knowledge.

**Methods:** We performed a search to identify all published studies on the association between hormonal contraceptive use and risk of CVST in women aged 15–50 years.

**Results:** Of 861 studies reviewed, 11 were included. The pooled odds of developing CVST in women aged 15–50 years taking OCPs was 7.59 times higher compared to women not taking OCPs (OR = 7.59, 95% CI 3.82–15.09). Data are insufficient to make conclusions about duration of use and other forms of hormonal contraceptives.

**Conclusion:** Oral contraceptive pills use increases the risk of developing CVST in women of reproductive age. Future studies are required to determine if duration and type of hormonal contraceptives modify this risk.

## Background

Cerebral venous sinus thrombosis (CVST) is a form of stroke whereby thrombosis occurs in the cerebral venous sinuses or veins. The incidence of CVST has been estimated at three to five cases per million population per year (1, 2). This represents about 0.5–1% of all strokes (1, 2). CVST affects the younger population (age <50 years) and is three times more common in women than men (1). Significant disability leading to dependency has been reported in about 5–10% of patients, and mortality rates range from 3 to 15% (3).

Several risk factors have been identified for CVST. These include medical conditions that increase the likelihood of thrombus formation such as thrombophilias, neoplasms, inflammatory conditions, transient situations (such as pregnancy, post-partum period, surgery, trauma, dehydration, CNS infections), and medications [such as oral contraceptive pills (OCP)] (1, 2, 4). Use of OCP has been shown in multiple observational studies to increase the odds of CVST by 5- to 22-fold (5). The presence of an inherited thrombophilia (such as Factor V Leiden or prothrombin-gene mutation) increases the odds further. Nonetheless, many of the previous studies were underpowered or had varying inclusion and exclusion criteria and did not adjust for the presence of other risk factors when evaluating the association between OCP use and CVST. Although a prior systematic review and meta-analysis were performed on this association, it was conducted over 7 years ago and did not make a distinction between those with thrombophilia and those without thrombophilia in the assessment of OCP use and risk of CVST (5). In addition, this review did neither evaluate information on type of OCP used and risk of CVST nor risk of CVST with the use of other hormonal contraceptives.

The objective of this systematic review and meta-analysis is to update available knowledge in regards to the association of CVST and OCP use among women aged 15–50 years. In addition, we reviewed the literature to determine if there was data on specific types of OCPs, duration of use, and other forms of hormonal contraceptives, such as transdermal patches, Depo-Provera injections, and intra-uterine devices, and the risk of developing CVST. Where possible, we tried to account for potential modifying and confounding variables when reporting on these associations and we used meta-regression techniques to explore heterogeneity among studies.

## Methods

### Search strategy

We performed a systematic review and meta-analysis following a predetermined protocol in accordance with the Meta-analysis of Observational Studies in Epidemiology (MOOSE) (6). We identified all potentially relevant articles using MEDLINE (from 1966 to June 2014), EMBASE (from 1980 to June 2014), Cochrane systematic review, the Cochrane Center for Clinical Trials, and CINAHL. All searches were carried out without any language restrictions, using free text and medical subject headings. We performed our search using two themes. The first theme included terms related to the exposure: hormonal contraceptives or oral contraceptives or OCP or birth control pill or ethinyl estradiol or desogestrel or levonorgestrel or Depo-Provera or Mirena IUD or Nuva-Ring or hormonal patch or Evra. The second theme included terms related to the outcome: cerebral-vein thrombosis or CVST or intracranial venous thrombosis or intracranial vein thrombosis or sinus thrombosis or dural thrombosis or dural venous thrombosis or dural vein thrombosis or venous infarct. Finally, we combined the terms from Theme 1 and Theme 2 with the Boolean operator AND. These terms were identified from exemplar articles identified in a scout search. Where appropriate, “permuted index” or “Tree/Thesaurus” was used to identify related terms including drug or generic names for hormonal contraceptives that could be included in the search. A theme was not used for study design in our initial search.

To identify articles that were yet to be cited or yet to be indexed in the electronic databases mentioned above, we handsearched major neurology and internal medicine journals published after December 2011 and the reference lists of all identified relevant publications. We also searched abstracts of major national and international Neurology, Internal Medicine, and Neurosurgery conferences from January 2009 until June 2014 using Conference Papers Index. In addition, we contacted local experts in stroke for information about other potential ongoing or unpublished studies. We also searched gray literature including unpublished theses and ongoing studies using the University of Calgary’s database at http://libguides.ucalgary.ca/greylit and the website http://clinicaltrials.gov/for unpublished clinical trials. Abstracts or papers in languages other than English were translated.

### Study selection

#### Inclusion and exclusion criteria

Articles were independently evaluated for eligibility using a two-stage procedure. Both primary authors (Farnaz Amoozegar, Bijoy K. Menon) initially reviewed all citations for original articles related to the primary study question in a broad sense. Inter-rater agreement was measured using the kappa statistic. Disagreements were resolved by consensus. Potentially relevant articles then underwent full text review. We included studies that met our explicit population, exposure, outcome, and design criteria. The study population was limited to women of reproductive age, specifically 15–50 years of age. Studies were excluded if the study population included: (1) pregnant women or post-partum women (up to 3 months after childbirth), (2) surgical patients, (3) patients hospitalized due to trauma, and (4) patients with prolonged immobilization. The exposure was any form of hormonal contraception, such as oral contraceptives pills, patches (Evra), or injections (depo-provera). The outcome was CVST, objectively confirmed by imaging: computed tomography (CT), magnetic resonance imaging (MRI), or angiography/venography. As CVST is a rare outcome, case–control studies were the most commonly employed study design to measure this association. However, cohort studies have also been performed in this area. Given that randomized controlled trials are not feasible, we limited our review to case–control and cohort designs.

#### Data extraction and study quality assessment

The same reviewers (Farnaz Amoozegar, Bijoy K. Menon) independently extracted data from all included studies. Disagreements were resolved by consensus. If missing data were identified for a given study, attempts were made to contact the authors to obtain the data. When multiple papers published the same data, we used the latest publication, and supplemented it, if necessary, with data from earlier publications.

Study quality was assessed based on the following criteria/questions:

(1) Were cases and controls well defined as per primary study hypothesis? Well-defined cases had objective confirmation of CVST and were 15–50 years of age, without autoimmune, neoplastic, or infectious diseases and not post-traumatic, pregnant, post-partum, or post-menopausal. Objective confirmation of CVST involved demonstration of thrombosis on one or more appropriate imaging modalities, such as CT venography, MR venography, or angiography. Well-defined controls were healthy individuals, between 18 and 50 years, and unrelated biologically to the patient.
(2) Were controls matched to cases? We defined this as being matched for at least one of: age, body mass index (BMI), smoking status, educational status, or ethnicity.
(3) Was there clear documentation of recent exposure to hormonal contraceptive use? This was defined as exposure to hormonal contraceptives within 2 weeks of CVST diagnosis for cases, or time of assessment for controls. Case–control studies reporting on exposure to hormonal contraceptives in the remote past may over-report exposure as an inherent recall bias (7); hence the reason we chose this as a criteria to assess study quality.
(4) Were potential confounders/modifiers addressed? Age, BMI, smoking status, hyper-homocysteinemia, Prothrombin-gene mutation, and Factor V Leiden) were the factors we looked for to address confounding and effect modification.
(5) Were *post hoc* statistical adjustments for confounders/modifiers performed before reporting on the exposure-disease relationship?

### Statistical methodology

The odds ratio (OR) was used as the common measure of association across studies. When studies only reported on stratum-specific OR for the relationship between the use of hormonal contraceptives and occurrence of CVST stratified by potential confounding variables, we calculated the assumed common OR across all stratifying variables using the Mantel–Haenszel method. Since the relationship between the use of hormonal contraceptives and occurrence of CVST may not have one true population estimate, primarily due to residual confounding by unknown genetic and environmental variables, we chose to derive pooled estimates using the Der-Simonian and Laird “random effects model.” To visually assess the OR estimates and corresponding 95% confidence intervals across studies, we also generated a forest plot.

A higher prevalence of hormonal contraceptive use in the population could potentially result in over-reporting of use. In addition, a higher prevalence of poverty, anemia, poor nutritional status, dehydration, and other residual confounding factors in developing countries may increase the incidence of CVST, when compared to developed countries. We therefore stratified all included studies by prevalence of hormonal contraceptive use and by developed/developing countries. We explored statistical heterogeneity by stratifying studies using “study quality” variables, prevalence of hormonal contraceptive use (> or ≤ median prevalence of OCP use in the control arm of all studies) and whether conducted in developing vs. developed countries. Statistical heterogeneity for the pooled OR was evaluated using the *I*^2^ statistic and the Cochran *Q* statistic.

In order to assess the nature of cumulative evidence over time on our research question, we performed a cumulative meta-analysis using the Der-Simonian and Laird random effects model. Finally, we tested for publication bias using Begg’s rank correlation test for small study effects (continuity corrected) and standard funnel plots. All *p*-values are two-sided, with *p* < 0.05 considered statistically significant. Analyses were performed using Stata/SE 12.1 software (StataCorp LP, College Station, TX, USA).

## Results

### Study identification and selection

**Figure 1:**
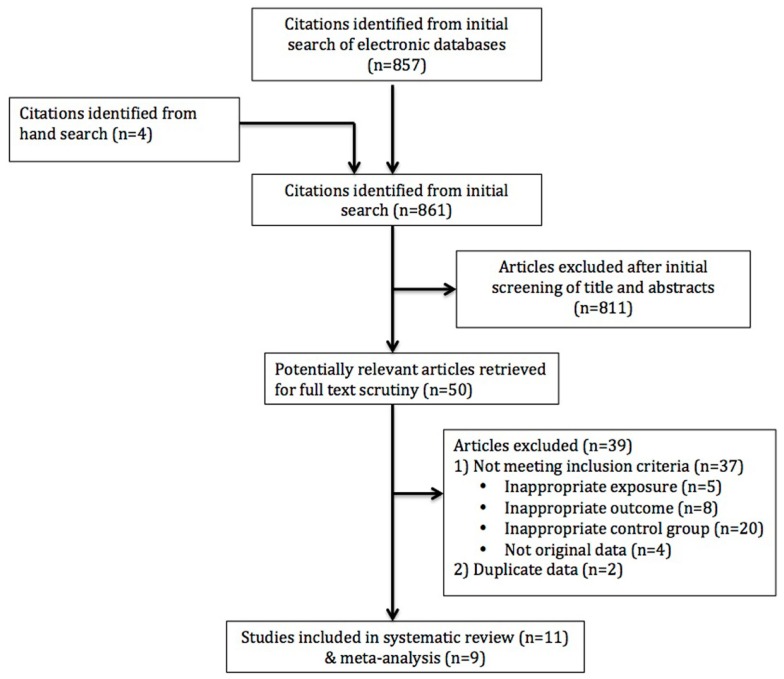
**Flow diagram for studies included in systematic review and meta-analysis**.

After excluding 148 duplicates, we identified 857 studies using our search strategy from MEDLINE (*n* = 249), EMBASE (*n* = 754), Cochrane systematic review (*n* = 2), and the Cochrane Center for Clinical Trials (*n* = 0) (Figure 1). An additional four studies were identified by handsearching the literature. The two reviewers agreed 94.4% of the time at this stage (unweighted kappa = 0.53). We excluded 811 articles after initial screening of titles and abstracts. Of the 50 studies selected for full text review, 11 studies were included in the systematic review (8–18). Of these 11 studies, only two studies reported on an association between the use of third generation OCPs and risk of CVST (12, 18). Nonetheless, data reported in these studies were such that we could not estimate an OR for CVST in women taking OCPs vs. those not taking them, i.e., relevant data were not available for a meta-analysis despite contacting the authors. Thus only 9 of 11 studies contained sufficient information for a meta-analysis to calculate the pooled odds of CVST. These nine studies were in the English language. Study period ranged from 1996 to 2005. All were case–control studies. Two studies were multi-center and three were from developing countries. Total subjects enrolled in the studies ranged from 32 to 2288. All studies reported only on the use of oral contraceptives. Only two studies reported on the duration of exposure to oral contraceptives. Characteristics of these nine studies are described in detail in Table 1.

**Table 1:** **Characteristics of studies included (*n* = 9)**.

Study (Author, year)	Study Setting	Cases (number)	Age range in years reported for cases	Cases (description)	Controls (number)	Age range in years reported for controls	Controls (description)	Type of hormonal contraception	Duration of exposure to hormonal contraceptives	Variables matched for *a priori*
Martinelli et al., 1996 (8)	Single center, Italy	16	21–64	First episode of CVT referred to the local thrombosis center; patients with neoplastic, autoimmune or infectious disease, pregnant and post-partum women were excluded.	48	not reported	Healthy controls with no history of thrombosis; no pregnant, post-partum women were included	Oral contraceptive only	Not reported	Age matched
Martinelli et al., 1998 (9)	Single center, Italy	25	15–64 (not reported after excluding pregnant, post-partum and post-menopausal women)	First episode of CVT referred to the local thrombosis center; none of them had neoplastic, autoimmune, or infectious disease; post-traumatic patients, pregnant and post-partum women as well as post-menopausal women were excluded	88	18–64	Healthy, biologically unrelated friends or partners of patients with no history of thrombosis; pregnant and post-partum women as well as post-menopausal women were also excluded	Oral contraceptives only	Cases (median = 15 months); controls (median = 26 months)	Age, geographic location and level of education
Reuner et al., 1998 (10)	Multi center, Germany	31	17–69	Patients with CVT aged 17–50 years; no data on whether patients with neoplastic, autoimmune, infectious disease were excluded; patients’ post-partum and in puerperium were excluded	148	18–63	Healthy blood donors aged 18–50 years	Oral contraceptive	Not reported	Age matched
de Bruijn et al., 1998 (11)	Multi- centre, Netherlands	40	18–54	Patients with CVT who participated in a treatment trial with heparin; younger than 18, pregnant and puerperium women were excluded	2248	18–49	Women aged 18–49 years from the continuous health interview survey of the Central Department of Statistic, Netherlands (pregnant women were not excluded)	Oral contraceptives only	Not reported	No
Martinelli et al., 2003 (13)	Single center, Italy	80	12–64 (includes male patients)	First episode of CVT referred to the local thrombosis center; patients with neoplastic, autoimmune, or infectious disease, post-traumatic patients, pregnant and post-partum women as well as post-menopausal women were not explicitly excluded	148	13–62 (includes males)	Healthy, biologically unrelated friends, or partners of patients with no history of thrombosis; no pregnant, post-partum women, or post-menopausal women were included	Oral contraceptives; other hormonal contraceptives mentioned	Not reported	Age matched
Cantu et al., 2004 (14)	Single center, Mexico	37	14–55 (includes male patients)	Patients with CVT admitted to hospital; no data on whether patients with neoplastic, autoimmune, infectious disease, patients’ post-partum, or in puerperium were excluded	66	16–53 (includes males)	Healthy controls of friends or relatives of other patients with neurological diseases who have no history of vitamin intake and no history of thrombosis	Oral contraceptive	Not reported	Recruitment directed toward young women
Rodrigues et al., 2004 (15)	Single center, Brazil	20	28 (median)	Consecutive patients with CVT; no data on whether patients with neoplastic, autoimmune, infectious disease were excluded; patients who were post-partum were however excluded	40	34 (median)	Women aged 20–50 years that came as outpatients for reasons other than thrombosis	Oral contraceptive only	Not reported	No
Ventura et al., 2004 (16)	Single center, Italy	14	19–44	Patients with CVT admitted to hospital; patients with neoplastic, autoimmune or infectious disease were excluded; no data on pregnancy, post-partum, or puerperium. Patients with use of OCPs <3 months prior to CVT were excluded	18	18–51 (including males)	Healthy volunteers with no history of thrombosis, cancer, cardiac, renal, liver, or hematological disorders and not taking any drugs or OCPs for at least 3 months prior to recruitment	Oral contraceptives only	Not reported	No
Gadelha et al., 2005 (17)	Single center, Brazil	19	3–46 (not reported for females)	Patients with CVT referred to the hemostasis lab of a tertiary university hospital, age >15 years and <50 years, pregnant and post-partum women excluded, major systemic diseases like cancer, diabetes, infectious or collagen disease, antiphospholipid syndrome; pregnant women or women in puerperium excluded.	134	15–62 (includes males)	Healthy controls accompanying patients to the same lab with no history of thrombosis or genetic relationship, age, and race matched	Oral contraceptives only	Not reported	Age Matched

### Study quality

Of the nine studies included, the quality ranged from moderate to high (Table 2). Four studies had well-defined cases, and five studies had well-defined controls. Controls were matched to case characteristics in five studies. Exposure to hormonal contraceptives within 2 weeks prior to CVST in cases and study assessment in controls was recorded in only three studies. Potential confounders and modifiers were discussed in five studies to varying extents. Variable adjustment for these factors occurred in the same five studies.

**Table 2:** **Study quality assessment. (*n* = 9)**.

Study (Author, year)	Well-defined cases^a^	Well-defined controls^a^	Matched controls^b^	Timing of exposure^c^	Adjustment for confounding variables^d^
Martinelli et al., 1996 (8)	Yes	Yes	Yes	Yes	Age
Martinelli et al., 1998 (9)	Yes	Yes	Yes	Yes	PT, FV
Reuner et al., 1998 (10)	No	No	Yes	No	None
de Bruijn et al., 1998 (11)	Yes	No	No	No	Age
Martinelli et al., 2003 (13)	No	Yes	Yes	Yes	Age, BMI, smoking, HyperHcy
Cantu et al., 2004 (14)	No	Yes	No	No	None
Rodrigues et al., 2004 (15)	No	No	No	No	None
Ventura et al., 2004 (16)	No	No	No	No	None
Gadelha et al., 2005 (17)	Yes	Yes	Yes	No	PT, FV

*A study is defined as being of high quality if it satisfies at least four of the five criteria above*.

*^a^Well-defined cases are objective confirmation of CVST in patients 15–50 years, without autoimmune, neoplastic, or infectious diseases and not post-traumatic, pregnant, post-partum, or post-menopausal). Well-defined controls are healthy individuals, between age 18 and 50 years, unrelated biologically to patient); Yes fulfills criteria for both or one of the above; “No” fulfills neither of the above criteria*.

*^b^Well-matched controls matched for at least one of age, BMI, smoking status, educational status, and ethnicity*.

*^c^Exposure within 2 weeks of CVST for cases or time of assessment for controls*.

*^d^Adjustment for confounding factors is defined as (1) Controls matched for at least one of age, body mass index (BMI), smoking status, educational status, and ethnicity and (2) adjustment during statistical analysis for age, BMI, smoking status, hyper-homocysteinemia, Prothrombin-gene mutation (PT) and factor V Leiden (FV)*.

*HyperHcy is hyper-homocysteinemia*.

### Effect of hormonal contraceptive use on occurrence of CVST

**Figure 2:**
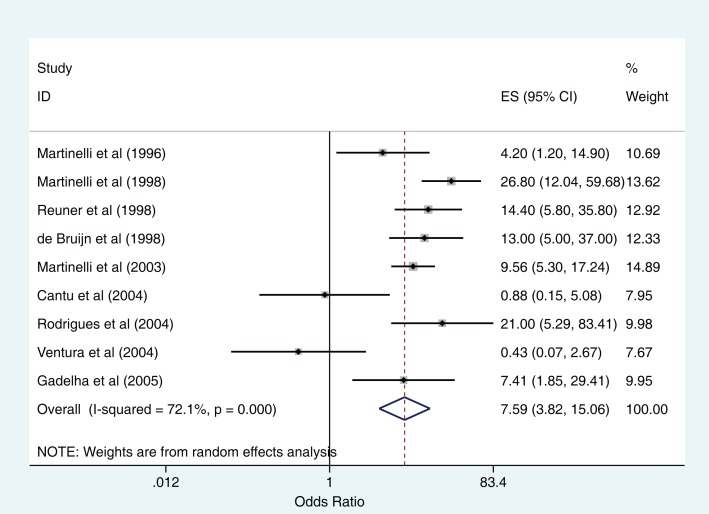
**Forest plot with pooled odds ratio for relationship between oral contraceptive use and cerebral venous sinus thrombosis (random effects model)**.

All 11 studies included in the systematic review show an association between the use of OCPs and CVST; use of OCPs increases the odds of developing CVST. Among the nine studies that reported OR, the pooled odds of developing CVST in women of reproductive age taking oral contraceptives was 7.59 times the odds of developing CVST for those not taking oral contraceptives (OR = 7.59, 95% CI 3.82–15.09, random effects model, Figure 2). The two studies that reported on the use of third generation OCPs suggested that the use of these newer generation OCPs was associated with an increased risk of CVST when compared to previous generation OCPs (12, 18). De Bruijn et al. assessed the risk of CVST with third generation OCPs and found an OR of 2 for risk of CVST with third-generation OCPs as compared to other OCPs (12). The actual number of healthy controls taking OCPs and third-generation OCPs were not available to us, despite contacting study authors. As a result, we were unable to calculate an OR for CVST with third-generation OCP use. Jick et al. used a cohort study design for assessing the incidence of CVST in users of four types of hormonal contraceptives (18). The incidence rate ratio (IRR) for CVST per 100,000 woman-years was 2.7 [95% confidence interval (95% CI) = 0.9–6.3], 1.6 (95% CI = 0.7–3.3), 0.7 (95% CI = 0.1–2.4), and 0.0 (95% CI = 0.0–4.8), respectively, in users of desogestrel, norgestimate, levonorgestrel, and the contraceptive patch. The incidence rate (IR) for non-exposed women was 0.4 per 100,000 woman-years (95% CI = 0.1–1.3). The authors, when contacted, had person time data for exposure and non-exposure; however, the study design was such that we could not calculate our summary estimate, i.e., the OR. In addition, original clinical records to confirm the diagnosis of CVST could not be obtained in this study.

**Figure 3:**
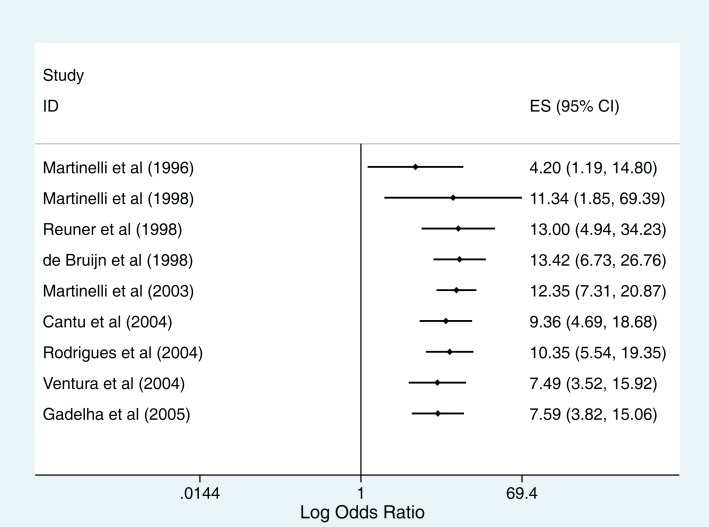
**Cumulative meta-analysis using the random effects model**.

Heterogeneity between the nine studies included in the meta-analysis was high (*I*^2^ = 72.1%, *p* < 0.001 for the Cochran *Q* statistic). Cumulative meta-analysis of these studies from 1996 to 2005 shows accumulating evidence over time of a positive association between the use of oral contraceptives and CVST (Figure 3). To explore reasons of heterogeneity between studies, we reported pooled ORs stratified by variables that were either “study quality” indicators or “potential confounders” (Table 3). Studies conducted in developed countries and those where the exposure was measured within 2 weeks of CVST showed higher odds of the outcome. Despite these findings, the confidence intervals around the estimates were wide. Furthermore, meta-regression did not identify any of the other variables as contributing to heterogeneity (Table 3).

**Table 3:** **Stratified analysis of pooled odds ratios with 95% confidence intervals (*p*-values generated from meta-regression)**.

	Odds ratio	95% CI	*p*-Value (meta-regression)
**Well-defined cases and controls^a^**			
Yes (*n* = 3)	8.26	3.95–17.25	0.84
No (*n* = 6)	5.73	0.79–41.49	
**Timing of exposure^b^**			
Within 2 weeks (*n* = 3)	11.15	4.44–27.98	0.51
Beyond 2 weeks (*n* = 6)	5.58	1.91–16.31	
**Prevalence of exposure^c^**			
>27.7% (*n* = 5)	6.4	1.99–20.57	0.82
≤27.7% (*n* = 4)	8.63	3.45–21.57	
**Countries**			
Developed (*n* = 6)	8.48	3.88–18.51	0.76
Developing (*n* = 3)	5.57	1.01–30.61	
**Adjustment for confounding factors^d^**			
Well done (*n* = 3)	13.15	6.06–28.52	0.35
Incomplete (*n* = 6)	5.05	1.72–14.87	

*^a^Well-defined cases are objective confirmation of CVST in patients 15–50 years, without autoimmune, neoplastic, or infectious disease and not post-traumatic, pregnant, post-partum, or post-menopausal). Well-defined controls are healthy individuals, between age 18 and 50 years, unrelated biologically to patient); Yes fulfills criteria for both or one of the above; “No” fulfills neither of the above criteria*.

*^b^Exposure within 2 weeks of CVST for cases or time of assessment for controls*.

*^c^Median prevalence of oral contraceptive use in the control arm of all studies*.

*^d^Adjustment for confounding factors is defined as (1) Controls matched for at least one of: age, body mass index (BMI), smoking status, educational status, and ethnicity and (2) adjustment during statistical analysis for at least two of: age, BMI, smoking status, hyper-homocysteinemia, prothrombin-gene mutation, or Factor V Leiden*.

### Assessment of publication bias

**Figure 4:**
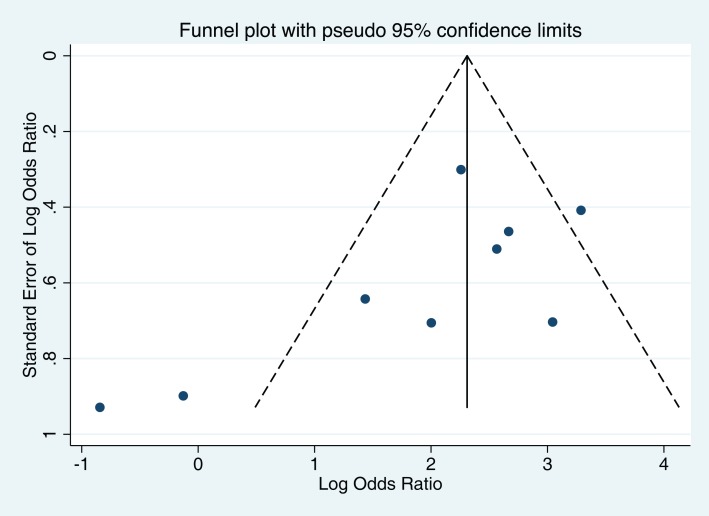
**Assessment of publication bias in meta-analysis using a funnel plot**.

A funnel plot of the log OR against the standard error of the log OR shows some asymmetry, with no studies in the lower right portion of the funnel and two small studies in the lower left portion outside the pseudo 95% confidence intervals (Figure 4). Despite a marginally significant Begg’s test (*p* = 0.048), it is unlikely that this plot suggests the presence of publication bias given the small number of studies included in the analysis and the type of studies that are absent in the funnel plot (small studies reporting a positive association between OCPs and CVST).

## Discussion

In this systematic review and meta-analysis, we found that the odds of CVST in women of reproductive age exposed to oral contraceptives was about sevenfold higher than the odds of CVST in those not taking oral contraceptives. This estimate is comparable to a previous meta-analysis 7 years ago (5). In addition, our review showed that there was insufficient evidence on the relative safety of third generation OCPs when compared to previous generation OCPs in increasing the odds of CVST. No data were found on the duration of exposure to OCPs and odds of CVST. In addition, the odds of CVST with OCP use is not significantly different in developing countries when compared to developed countries. Our review also showed that the odds of CVST with the use of “other hormonal contraceptives” is largely unknown. These latter issues were not assessed in detail in the prior systematic review.

We found moderate to high heterogeneity between studies included in our meta-analysis. Given varying quality of studies included in our meta-analysis, study quality itself could be responsible for this heterogeneity. We did not, however, find any statistical evidence that study quality was responsible for this heterogeneity. Variables such as age, smoking, and prothrombotic genetic mutations can certainly contribute to the risk of CVST and influence the results obtained (3, 5). Meta-regression, however, did not identify these variables as contributing to study heterogeneity either.

The prevalence of CVST is reported to be high in developing countries when compared to developed countries (4, 19). However, we did not find a statistically significant increase in the odds of CVST with OCP exposure in the developing countries. Our results lead us to speculate that the independent biological effects of OCPs on thrombosis may be stronger than “effect modification” of the relationship between OCP use and risk of CVST by factors such as malnutrition, dehydration, or anemia. A caveat to this conclusion would be that none of our included studies were from Sub-Saharan Africa or South Asia, regions of the world where malnutrition and anemia are more prevalent (20, 21). This could be because of publication bias or because no studies of sufficient quality have yet been published from these regions. We did find minor statistical evidence for publication bias in our study. Begg’s test does not tell us as to the direction of this bias. Interestingly, the funnel plot (Figure 4) shows absence of small studies reporting on a positive association between use of OCPs and CVST. We cannot comment if such studies from the above-mentioned regions failed to cross the publication threshold, but that seems less likely. We do feel that there is a need for high quality studies from these regions of the world.

We only found two studies reporting on the risk of CVST with the use of third generation OCPs (12, 18). Third-generation oral contraceptives are combined pills containing either gestodene or desogestrel that were developed to reduce risk of cardiovascular disease due to their reduced androgenic activity (12). There is controversy as to whether third generation OCPs are associated with increased risk of venous thrombosis when compared to second generation OCPs (22, 23). Biological basis of the increased risk could be a differential effect on various coagulation factors, resulting in overall increased thrombogenicity (24, 25). Proponents for the use of third generation OCPs point out various biases including a healthy user bias, recent introduction bias, prescribing bias, and referral bias, resulting in increased reported risk with these OCPs when compared to previous generation OCPs (26, 27). Other studies, including a detailed meta-analysis refuting these biases, suggest that third generation OCPs put women at higher risk of developing venous thrombosis (22, 28). Our review of the literature to the current time leads us to conclude that no definite recommendations can be made in favor of the safety of third generation OCPs when compared to previous generation OCPs in women with risk factors for developing CVST. Furthermore, in such women, non-hormonal contraceptive methods are preferred (26, 29, 30).

We found no major studies assessing the risk of CVST with the use of other hormonal contraceptives, such as contraceptive rings or transdermal patches. Nonetheless, our search did reveal a couple of case reports on CVST with the use of hormonal contraceptive rings (31, 32). In addition, a recent systematic review reports a high risk of venous thrombosis (CVST not assessed) with the use of these non-oral hormonal contraceptives (33). In our opinion therefore, unless there is new evidence to the contrary, these types of contraceptives should also be avoided in women with risk factors for CVST.

Our study has limitations. Biases inherent in a case–control study design can influence our summary estimate (34). Even though we explored the role of potential confounders in interpreting a pooled estimate of the measure of association between hormonal contraceptive use and CVST, residual confounding by variables not studied or unknown could always exist (34). An interaction between risk factors for CVST and hormonal contraceptive use was not reported in most studies and therefore could not be explored in detail. Nonetheless, despite the limitations of doing a meta-analysis of case–control studies, our study has several strengths. First, CVST was objectively confirmed in all studies. Second, we can conclude with confidence that the odds of CVST is increased with the use of OCPs as compared to individuals not using OCPs; the studies in this area consistently show this association. In addition, our cumulative meta-analysis demonstrates that this association holds true and strengthens with time. This association also has biological plausibility (2, 4).

Part of the issue in studying CVST is the rarity of this disorder and the inability to study it in large cohort studies or randomized controlled trials (3). Therefore, case–control studies remain the most feasible design in studying CVST. Future studies should strive to improve the description of the association in question, and future researchers in this area should be mindful of the appropriate population to study. Variables that increase the risk of CVST, such as pregnancy or the post-partum period, prothrombotic genetic mutations and head trauma should be excluded in both cases and controls. Controls should be matched to cases by age, BMI, socioeconomic status, etc. Duration and type of hormonal contraceptive use and its timing in relation to CVST (for cases) or study assessment (for controls) should be documented and then analyzed in the results. Finally, analysis of results should include an overall estimate of effect, but also subgroup analysis to look at variables such as type of hormonal contraception, duration, and timing of use.

## Conclusion

Oral contraceptive use increases the risk of CVST in women of reproductive age sevenfold when compared to those not using this method of contraception. This systematic review suggests the need for future studies to answer whether duration and type of hormonal contraceptive use modifies the risk of developing CVST. However, based on our review of the literature, we would recommend that women with other risk factors for thrombosis, such as smoking, immobility, history of thrombosis or thrombophilia, or a past history of CVST, choose alternative non-hormonal methods of contraception.

## Conflict of Interest Statement

The authors declare that the research was conducted in the absence of any commercial or financial relationships that could be construed as a potential conflict of interest.
